# The Unreliability of Online Review Mechanisms

**DOI:** 10.1007/s10603-022-09514-7

**Published:** 2022-04-12

**Authors:** M. Narciso

**Affiliations:** Faculty of Law, Maastricht University, Maastricht, Netherlands

**Keywords:** Online reviews, Online platforms, EU consumer law, Consumer information

## Abstract

Online reviews have an undeniable impact on the market and are an important source of consumer information. From a legal perspective, online reviews actively influence consumers’ decisions to enter into a contract. Moreover, online reviews convey pre-contractual information that consumers find relevant and easy to understand, unlike the pre-contractual information disclosed as a result of EU law–based information duties. From this perspective, online reviews could potentially be seen as a complement of the flawed EU law–based information paradigm and regulatory improvement options based on reviews could be explored. However, the unreliability of online reviews is an obstacle that haunts consumers, practitioners, regulators, and academics alike. This unreliability has previously been identified as a reason not to award online reviews a more significant role in the EU law–based regulatory framework of pre-contractual information in consumer contracts. This paper explores the merits of this argument by discussing how the unreliability of online reviews is currently regulated. This paper takes a broad perspective on regulation, focusing not only on EU consumer legislation, but also looking at standardization, soft law, self-regulation, and the role of national consumer authorities. Overall, this paper argues that there are sufficient measures in place to shift the debate from the unreliability of reviews to reviews’ potential role in the protection of consumer informational interests.

## A Legal Approach to Online Reviews

Online review mechanisms allow platform users (both businesses and consumers) to share their feedback on a certain product or service they have acquired. This definition is broad and includes reviews in the form of a written text (including Q&A forums), a numerical rating (for example, from 1–5), a video (including videos posted on social media platforms), or visual elements (such as emojis). At their core, online review mechanisms facilitate electronic word-of-mouth, which has been defined in the context of sales as any (online) statement made by consumers about a product or service that derives from their experience (King et al., 2014, p. 167; Lis & Neßler, 2014, p. 63). While online reviews are not a recent phenomenon, the rise of the platform economy in recent years turned them into a globalized factor reaching an unprecedented number of individuals. The current impact and importance of online review mechanisms not only in the conclusion of consumer contracts but also in the market in general cannot be ignored. Studies show that over 90% of consumers read reviews before making a purchase^1^ and that over 90% of travellers consult online reviews when planning a trip (European Commission, 2014a; 2014b, p. 10). A 2015 study by the UK’s Competition and Markets Authority showed that an estimated 23 billion pounds of online shopping are influenced by online reviews (Competition & Markets Authority, 2015, p. 3).

From my perspective, online reviews have two main functions: signalling trust (or building reputation) among market players and providing users with pre-contractual information. Both functions are equally relevant from a legal perspective, albeit the latter has unfairly deserved less attention than the former. Online reviews can be seen as a practical, accessible alternative or complement to the long-worded and often complex terms and conditions or mandatory disclosures. If understood as pre-contractual information, online reviews can help overcome several of the shortcomings faced by the much-discussed information paradigm in EU consumer law. The imposition of information duties on professional parties as a means of consumer protection frequently faces severe (and justified) criticism, such as imposing an information overload on consumers or the fact that consumers cannot understand the complex and often unclear technical language used. The regulation of information by EU consumer law is also criticizable, as it frequently imposes duties to inform in a “clear and comprehensible manner” (such as in Article 5 and 6 of the Consumer Rights Directive) or similar formulations and it refrains from clarifying the meaning of such open-ended terms. Recently, I have been exploring how robust, varied, and easily accessible online reviews can be a solution to these problems. However, online reviews face a shortcoming of their own: their unreliability, or their occasional inability to inform consumers of the *real* experiences of other consumers. As a consequence, scholars have called for a regulation of online review mechanisms (Busch, 2016; Ranchordás, 2018). Indeed, this unreliability raises questions regarding the regulatory role that reviews can play. Considering the potential of online reviews to affect consumer choices, for reviews to perform any role in the public regulation of consumer contracts their reliability must naturally be assured or, at least, addressed. Still, online reviews remain relatively unexplored from a legal perspective. The rare legal literature on it highlights their unreliability as an obstacle, but the efforts made in tackling that unreliability have not been significantly discussed. These efforts took centre stage when, in 2019, the Directive on the modernization of consumer protection rules—also known as the Omnibus Directive—became the first EU legislative instrument to explicitly regulate online review mechanisms. While previous research addressed how EU consumer law generally applies to online reviews–related practices (Narciso, 2019), it is still unclear to what extent the Omnibus Directive offers additional protection against reviews’ unreliability. Furthermore, online reviews are regulated by actors and instruments beyond those found in EU or national legislation.

The question therefore arises: to what extent is online reviews’ unreliability addressed by existing regulation, and to what extent does it pose an insurmountable obstacle to online reviews’ possible regulatory role(s)? To answer these questions, this paper begins by framing reviews’ unreliability as a problem for not only consumer protection but also for the functioning of the internal market in general (Sect. 2). To illustrate the added value of advancing the discussion surrounding online reviews beyond their unreliability, this paper briefly discusses online reviews’ role as pre-contractual information and contrasts it with the shortcomings of the EU law–based information paradigm (Sect. 2). Then, this paper analyses the currently existing measures that deal with the unreliability of online reviews from a broad regulatory perspective (Sect. 3), focusing not only on EU consumer law (including the Omnibus Directive), but also on self-regulatory efforts by online platforms, soft law, and enforcement initiatives by national consumer authorities. Section 4 concludes. Overall, this paper argues that there are sufficient measures in place to at least advance the debate from the reliability of reviews to reviews’ potential role in the protection of consumer interests.

## Online Review Mechanisms and Consumer Contracts

While legal research on online reviews is rare, non-legal literature is abundant (among many, Calheiros et al., 2017; Einav et al., 2016; Filippas et al., 2018; Guo et al., 2017; Korfiatis et al., 2012; Luca, 2016). The existing literature mainly discusses online review mechanisms as reputation systems, as well as their benefits and shortcomings in market regulation from an economic perspective (for example, Einav et al., 2016). A reputation system is mainly translated into quality cues, a signal that indicates whether the potential contracting partner is reliable. As a reputation-signalling mechanism, online reviews have a considerable impact on the market. For example, online reviews allowed individuals, small and micro businesses to enter a previously hard-to-access market, since they to some extent replaced advertising and therefore lowered market entry costs (Einav et al., 2016; Luca, 2016). By signalling reputation, online reviews also allow consumers to sanction bad behaviour by professional parties, which provides an incentive for compliance and reduces the risk of moral hazard. Additionally, the positive or negative character of online reviews has a direct impact on consumer choice and, consequently, on businesses. For example, it has been shown that a one-star increase on Yelp causes a 5–9% increase in revenue (Luca, 2016, p. 2). Studies also show that a 10% increase in travellers’ reviews leads to a 5% increase in bookings (Ye et al., 2011, pp. 634–639). Online reviews (or the lack thereof) can also negatively affect job opportunities of workers. For example, research has shown that the number of completed tasks on TaskRabbit is positively correlated with the number of reviews (Hannák et al., 2017, p. 1921). The market impact of online reviews highlights the negative consequences that unreliable reviews can bring to consumers, businesses, workers, and employers, as well as the challenges potentially posed to consumer law, general contract law, competition law, or labour law.

### Lack of Reliability

It has been widely confirmed that online reviews can be unreliable (among many, Einav et al., 2016; Filippas et al., 2018; Mayzlin et al., 2014). This unreliability was the main reason behind legislative regulation of online review mechanisms at EU level—as is acknowledged in Recital 49 of the Omnibus Directive—and naturally so. The existence of unreliable online reviews means that consumers may, at times, base their decision to contract on equivocal information. Online reviews’ unreliability can result from different aspects. In particular, it is possible to distinguish between four types or sources of unreliability: lack of reviews, biased reviews, fake reviews, and platforms’ presentation, processing, and ranking practices. As will be explained in Sect. 3, these different types or sources of unreliability can lead to different legal consequences.

Regarding the first, it is well known that not all consumers leave a review after they experienced a product or a service. When paired with the fact that platforms or traders often do not disclose how many consumers leave a review after a transaction versus how many do not, this makes it evident that the lack of reviews can be misleading. Consumers might assume that the information they are getting is representative of most experiences, when, in reality, it might only represent extreme cases or extreme opinions. Moreover, consumers may not realize that the opinion they are seeing represents only a small portion of the total transactions. Other problems related to the lack of representativeness of online reviews have been pointed out by the literature. For example, Edelman suggests that there are several levels of non-representativeness, such as the systematic inability of certain products or groups of products (like “boring products”) to gather reviews (Edelman, 2017, p. 638).

Additionally, online reviews can be biased. This can happen if their writer is affected by, for example, fear of retaliation from the reviewed party or a wish for social desirability. Biased reviews can lead to a problem known as reputation inflation. Nosko and Tadelis (2015) showed that the median seller on eBay has a score of 100% positive feedback ratings. In addition, research has shown that when private feedback options are available (that is, feedback that is not shown to the public/users or to the reviewed business), they often contradict the public feedback of the same experience. For example, Filippas et al. (2018) showed that almost 30% of users who privately claimed to have had a negative experience gave the highest scores on public feedback.

Online reviews are also unreliable when they are not based on a real experience but are rather written with the purpose of, for instance, intentionally promoting a business or harming another business’ reputation (fake reviews). While the literature and existing regulation address them as the most concerning issue in this context, it is not clear how common fake reviews are. There are contradicting reports on this. Studies have suggested that 16% of Yelp’s reviews were potentially fake (Luca & Zervas, 2016). On the other hand, TripAdvisor recently revealed that in 2018 a “mere” 2% of reviews were deemed fake (TripAdvisor, 2019, p. 6). Interestingly, TripAdvisor revealed that 73% of these fake reviews were blocked before they were posted, which means that, in practice, they did not harm or mislead consumers. Similarly, Trustpilot recently revealed that it removed over 2.2. million fake or harmful reviews from their platform in 2020 (Trustpilot, 2021, p. 26). It is important to note in this context that consumers seem to be increasingly aware of the potentially fake nature of some online reviews, which may mean that they are more rigorous when deciding which reviews to trust. For example, research has shown that 81% of consumers believe they have read at least one fake review in the previous year.^2^ Accordingly, numbers show that the majority of (peer) consumers have reservations about the reliability of online reviews and their ability to “provide adequate information, safety and protection” (European Commission, 2014a, p. 10; European Commission, 2014b, p. 57). Online platforms also take frequent action against fake reviews, particularly as a response to the several businesses that nowadays focus on providing fake reviews against remuneration, as a reputation-management service.^3^ Recently, Amazon conducted a large purge of fake reviews from its platform: According to the Financial Times, Amazon deleted approximately 20,000 product reviews written by some of its top 10 UK reviewers.^4^ In this particular case, Amazon’s action followed an investigation conducted by the Financial Times, which revealed that several reviewers were profiting from posting (allegedly paid-for) positive reviews.

In addition to the potentially unreliable nature of online reviews themselves, platforms’ practices—such as algorithms and rankings related to online reviews—can also raise reliability concerns. The algorithm behind the presentation and ranking of online reviews is defined by the online platform, and it is often not made (completely) public to its users. For example, TripAdvisor does not disclose the specific factors that influence the ranking of online reviews but merely states that the review’s level of helpfulness or relevance (as decided by consumers) is not taken into account for such purposes.^5^ This lack of transparency means that consumers are not aware of what exactly determines the selection and ranking of the online reviews they read. It is important to note that not all literature agrees that transparency is necessary to fight biased algorithmic decision-making (on this topic, and defending that transparency is necessary, see Kim, 2017). But this lack of transparency also means that, in an extreme case, an online platform can “secretly” determine that only positive reviews are shown, or that (positive) reviews on businesses that have paid for promotion appear first. Additionally, it is often unclear how online reviews influence the ranking of offers presented to consumers. Illustratively, research shows that online travel agents such as Booking.com have incentives to present their offers ranked not according to reviews or to the needs of the consumer, but to the prices charged by the hotels on other platforms (Hunold et al., 2018). It is noteworthy that manipulated, biased, or unreliable reviews may affect several parties involved: the intermediary online platform because consumers lose trust in it; the business, because a negative fake review lowers its revenue; and the consumers, because a positive (fake or biased) review may deceive them into acquiring a product or a service that does not match the advertised characteristics. It should also be highlighted that, from a legal perspective, while online reviews are generally discussed in the context of consumer protection, businesses also need to be safeguarded from the consumers’ ability to make or break their reputation via fake negative reviews. In fact, there are multiple reports of consumers threatening to leave a negative review in case they do not get a benefit, such as a price discount.^6^

### Online Reviews as Pre-contractual Information

As previously stated, online review mechanisms go beyond reputation-building. Online reviews do not merely signal whether the contracting partner is trustworthy, but they also convey information about several aspects relevant to the possible conclusion of a consumer contract, such as subjective and objective assessments of the characteristics of the product, information on the quality of the product, or information on the possible use of the product (Ghose & Ipeirotis, 2006, pp. 303–310; Jin et al., 2016, p. 64; Thakur, 2018, p. 48). In fact, online reviews allow access to other users’ previous experiences, which renders the information gap smaller and allows consumers to better contextualize information based on their own needs. The difficulty in contextualizing pre-contractual information is one of the main problems that consumers face when concluding contracts, which is particularly concerning in contracts that entail a high financial burden (such as expensive travel packages or credit agreements).

The information paradigm in EU consumer law—that is, the protection of consumers through the frequent imposition of duties to inform on the professional party—has been severely criticized. The protection-through-information approach is a trait of EU consumer Directives across all fields: the Consumer Rights Directive, the Package Travel Directive, the Unfair Commercial Practices Directive, the Consumer Credit Directive, the Mortgage Credit Directive, among many others. When complied with, these information duties are typically translated into terms and conditions or (long) pre-contractual standardized forms (such is the case in credit agreements, for example). Recurrent critiques to the information paradigm include the fact that, as a result of EU legislation, consumers are overloaded with information (Kästle-Lamparter, 2018; Straetmans, 2019, pp. 4–5; van Boom, 2011, p. 360; p. 395). Consumers’ lack of interest in reading terms and conditions has been well reported in the literature (Elshout et al., 2016). Additionally, consumers often cannot understand the information disclosed, since it is often too complex, highly technical, or obscure (Seizov & Wulf, 2020, p. 158). On the other hand, however, consumer interest in online reviews is rising. Not only are more consumers consulting reviews more regularly (Trustpilot, 2021, p. 5), but the trend in the submission and influence of online reviews is increasing (Mariani et al., 2019). In this sense, online reviews are not only a rich source of pre-contractual information, but they also play, at least from a practical perspective, a complementary regulatory role regarding EU consumer law. In other words, both sources of information simultaneously contribute to the consumers’ decision to enter a contract. Online reviews’ nature as a bottom-up alternative to a top-down approach of information regulation seems promising in terms of succeeding to deliver consumers the information that they are indeed interested in. In fact, from a substantive perspective, most of the information conveyed through online reviews is generally not conveyed to the consumer as a result of EU law–based information duties. For example, EU consumer law imposes duties to disclose an objective rather than a subjective or use-based description of the characteristics of the product in question, which typically also includes the disclosure of the price, costs, and related aspects, as well as the legal rights of consumers. These conclusions can be extracted with certainty from an analysis of the hundreds of information duties scattered across EU consumer law. For example, all these aspects are considerably covered by the main information provisions in the Consumer Rights Directive (Articles 5 and 6), in the Consumer Credit Directive (Articles 5 and 6), the Package Credit Directive (Article 5), and the Mortgage Credit Directive (Articles 11, 13, and 15). However, research has demonstrated how subjective, experience-based information (such as, but not limited to, advice and recommendations) is preferred by consumers over objective, factual information (for example, Schaffner et al., 2015). This type of information is typically conveyed in online reviews. While this path remained so far unexplored in the literature, I believe that online reviews can be used to, among other goals, re-evaluate the benefits and (most of all) the shortcomings of EU consumer legislation in the regulation of pre-contractual information. In this sense, for example, online reviews can be useful as a source of behavioural data on consumer preferences, which the legislator can use to reduce, expand, or adjust existing information duties. Moreover, the very existence of online reviews as a solid and structured source of pre-contractual information could in theory justify a deregulation of consumer information by the EU legislator, at least in some areas.

Conceptualizing online reviews as pre-contractual information is not an easy task, partly due to the heterogeneity of online review mechanisms. Indeed, understanding the informational role of online reviews in consumer contracts depends on understanding their heterogeneity. It is misleading or inaccurate to claim that all online review mechanisms convey the same pre-contractual information. Different review mechanisms put in place by different platforms mean that consumers will receive different information according to the contract they conclude and, most importantly, according to where they have read reviews. One example of such heterogeneity can be seen in the distinction between professional reviews and user reviews (Zhou & Duan, 2016, pp. 204–206). While both are relevant sources of consumer information, they not only differ regarding their origin but also their content. Professional reviews are provided by experts “to build up the product reputation, offer product information, and serve as indirect advertisements” (Thakur, 2018, p. 50). Professional reviews or third-party reviews focus on product-attribute information (e.g., performance of the product) and the final assessment given by the reviewer tends to be correlated with the performance of these attributes (Chen & Xie, 2008, p. 480; Zhou & Duan, 2016, p. 203). User reviews, on the other hand, are based on the user’s experiences with the product or service and they tend to focus on “whether and how a product matches a specific individual’s preference and usage condition” (Thakur, 2018, p. 50; Zhou & Duan, 2016, pp. 203–204). Even within user (or consumer)-based review mechanisms, the specific design and the specific measures implemented by each review mechanism vary greatly. For example, Amazon’s review mechanism differs from Trustpilot’s review mechanism. Notably, the object of reviews is different: While Amazon’s reviews are directed towards an assessment of a product acquired from Amazon or from another business, Trustpilot focuses on reviews of the business itself. Then, while Trustpilot allows reviews from users who have not concluded a transaction but have had some type of contact with the reviewed business, Amazon asks for post-purchase reviews only. The type of information resulting from each platform will naturally differ. While exploring the legal consequences arising from this heterogeneity falls outside the scope of this paper, this exploration deserves further research by the literature. Conceptualizing online reviews as pre-contractual information also faces other apparent obstacles, such as the fact that this pre-contractual information originates from a third party to the consumer contract (i.e., from other user-consumers). This, however, does not stand in the way of perceiving online reviews as pre-contractual information, if pre-contractual information is understood as all information that is disclosed to or acquired by consumers before they enter a contract, regardless of its source. A different question would be whether, considering their nature as pre-contractual information, the existence or provision of online reviews could justify traders’ compliance with existing pre-contractual information duties stemming from EU consumer law. Answering this question would require a deeper analysis of, for example, the level of control exerted by online platforms or business-retailers over their online review mechanisms. Exploring the contract law consequences of such a classification also falls outside the scope of this paper, but these are nonetheless noteworthy points, as they show how perceiving online reviews as pre-contractual information can impact the interpretation of legal rules.

As mentioned earlier, online reviews’ unreliability has been pointed out as one of the reasons why they cannot assume a more acknowledged (if not prominent) role in the regulation of consumer information. In fact, the evaluation of the Consumer Rights Directive explored (albeit superficially) “whether review and rating systems can possibly replace some of the functions of the current consumer acquis” (European Commission, 2017, p. 56). In particular, in an online survey for the study on the Consumer Rights Directive, stakeholders were asked whether “consumer information requirements [are] less relevant when platforms operate review and rating systems” (European Commission, 2017, p. 56). The stakeholders’ answers are not, unfortunately, mentioned in the report, and the EU did not follow up on this. The Omnibus Directive, for example, does not refer to online reviews in their pre-contractual information capacity, and it does not decrease existing information duties; on the contrary. The evaluation of the Consumer Rights Directive (2017, p. 57) explains that previous studies showed that consumers do not consult reviews *systematically*, which meant that “(…) ratings and reviews are unlikely to reflect the experience of all or most platform users.” Additionally, the evaluation of the Consumer Rights Directive warns that online review mechanisms “(…) risk to give a misleading or inaccurate impression to platform users.” As can be seen, unreliability was an insurmountable obstacle for the Commission to consider the information conveyed by online review mechanisms as replacements of information duties. Nevertheless, the evaluation of the Consumer Rights Directive mentions that online review mechanisms can be considered a “supplementary tool to enhance trust” and highlights the need to step up “enforcement efforts against fake reviews and misleading practices.”In the next section, I will explain how existing regulatory efforts tackle concerns regarding online reviews’ unreliability.

## Regulation of Online Review Mechanisms

Several regulatory actors have developed rules that apply to unreliable online reviews, although the main focus is on fake reviews and on their presentation to consumers, and understandably so. This is true not only for EU legislators, but also for the platforms themselves, academic groups, and national consumer authorities. It is noteworthy that the feasibility of regulatory action to guarantee online reviews’ reliability varies according to the source or type of unreliability (discussed above, in 2.1.). For example, regulating biased reviews is rather difficult, since biased decisions are inherent to human behaviour and, more than that, EU consumer law does not apply to consumer actions, but generally to traders’ actions. In this sense, EU consumer law will prove beneficial mainly when it comes to fighting fake reviews written or ordered by traders, as well as to presentation and ranking practices by platforms. In addition, when assessing the legal framework surrounding unreliable online review mechanisms, it is important to keep in mind that the different types of unreliability can also dictate different legal consequences (such as from a contract law or criminal law perspective). For example, writing a fake review demonstrates an intention to deceive, which may be absent from an inadvertently biased review. Depending on the legal system in question, the presence of the intention to deceive determines the existence of fraud. In this section, I explain the efforts made by regulators and by platforms themselves to improve online reviews’ reliability.

### EU Consumer Law

Online reviews fall within the scope of several EU Directives, in particular of the Unfair Commercial Practices Directive and of the E-Commerce Directive. Recently, through the Omnibus Directive, the EU explicitly regulated, for the first time, online review mechanisms. The Omnibus Directive and the explicit regulation of online review mechanisms are the results of a process that started almost 10 years ago. Indeed, the first study requested by the European Commission that covered online review mechanisms was conducted in 2012. The results were published in 2013 (European Commission, 2014b). In May 2017, the European Commission released its results on the fitness check of consumer and marketing law, as well as on the evaluation of the Consumer Rights Directive. Furthermore, in the context of the 2018 “New Deal for Consumers,” the European Commission published a behavioural study on the transparency of online platforms (European Commission, 2018). As a response to these studies and consultations, in April 2018, the EU Commission announced the “New Deal for Consumers,” a legislative package composed of two proposed Directives: the Directive on the modernization of consumer protection rules (the Omnibus Directive) and the Directive on representative actions for the protection of the collective interests of consumers. In what concerns online review mechanisms, the Omnibus Directive amended the Unfair Commercial Practices Directive. Finally, the recent Regulation on promoting fairness and transparency for business users of online intermediation services (P2B Regulation, proposed in 2018 and approved in 2019) is also relevant in the context of review mechanisms, even if marginally. This legislative instrument directly regulates neither online review mechanisms nor business-to-consumer relations, but it deals with the importance of rankings of offers of goods and services by providers of online intermediation or search services and their impact on consumer choices. The rules applicable to unreliable online reviews and related practices in these four instruments will now be analysed. It is noteworthy that the proposed Digital Services Act—at the time of writing of this article, undergoing discussions between European Parliament and Council—will also be extremely relevant for the regulation of online review mechanisms. Indeed, the proposed Digital Services Act, if passed as legislation, will deal with the role of platforms and other actors in preventing, identifying, and removing illegal online content, which can cover fake or defamatory online reviews, as well as with transparency obligations on recommender systems and dark patterns, which will tackle misleading practices surrounding the presentation and processing of online reviews. However, since this proposed Regulation has not been approved by the EU co-legislators, it will not be analysed in detail in this paper.

#### The Regulation on Promoting Fairness and Transparency for Business Users of Online Intermediation Services

As said, the P2B Regulation on fairness and transparency regulates the ranking of offers by intermediary platforms or search engines. Articles 5(1) and (2) of the Regulation determine that providers of online intermediation services and online search engines provide information about the main parameters that determine their ranking mechanisms. According to Article 2(8) and Recital 24 of the Regulation, “ranking” includes the presentation of offers that result from, among other aspects, rating or review mechanisms. The Regulation intends to promote and strike a balance between fairness, predictability, and transparency of such ranking mechanisms (Recital 8). Moreover, according to Recital 25, indicators used to measure the quality of goods can be considered as “main parameters” of a ranking, which means that, when online reviews and ratings are such an indicator, these mechanisms must be described to the corporate user in plain and intelligible language. In this sense, providers of intermediation services should make it clear when and how online reviews impact rankings. It is important to highlight, however, that providers of online intermediation services or online search engines will not be obliged to disclose the detailed functioning of their ranking systems, including algorithms (Article 5(5)). Platforms are only under the duty to provide “sufficient” descriptions to enable business users to “obtain an adequate understanding” of the ranking mechanism. However, the disclosure of the main parameters behind the ranking—including online review mechanisms—should include an explanation on whether business users can change their ranking (e.g., to rank higher) against remuneration (Article 5(3)). Information on the possibility to rank higher on a review-based system or platform—such as Booking.com—is also important for consumers, since it might be unexpected to find lower-rated businesses ranked higher than higher-rated businesses, especially if the platform in question is particularly known for providing online reviews and ratings. In this sense, these obligations are relevant for business users—because it allows them to adjust their offers according to the ranking system in place—but also for consumer users—because it allows them to compare offers more easily, and to understand why certain offers rank higher. Booking.com, for example, discloses that Trip Providers (business users) can pay a commission to increase their ranking within the platform.^7^ While these transparency or information rights within a business-to-consumer contract do not arise from this Regulation, they stem from the regime of the Unfair Commercial Practices Directive, as will be explained below.

#### The E-Commerce Directive

In broad terms, the E-Commerce Directive establishes a general rule of exclusion of liability for an online platform that hosts illegal online reviews (Article 14), which applies unless the online platform has knowledge of the illegal character of the review or if it does not remove it quickly enough. Illegal reviews can be, for instance, those obtained through blackmail or reviews that constitute fraud under national laws, which, in most cases, will be fake reviews. Besides, the exemption of liability does not apply in case the online platform plays an active role in the storing and processing of online reviews. Finally, the E-Commerce Directive establishes that Member States cannot impose any duties on the hosting online platform to monitor or fact-check information transmitted or stored, such as online reviews (Article 15). While this Directive attempts to create a balance between self-regulation and top-down regulation, when it comes to online reviews, it achieves neither one successfully. The existing provisions in the E-Commerce Directive create incentives for online platforms to detach themselves from any active monitoring of fake or misleading online reviews since an active role can mean the inapplicability of the exemption of liability. This is not ideal, since online platforms are in the best regulatory position to control the origin and reliability of the reviews posted by their users. The European Commission has explained that carrying out ancillary services such as managing online review mechanisms should not prevent the application of the exemption of liability, since this interpretation would be an incentive towards “responsible behaviour by all types of online platforms in the form of voluntary action, for example to help tackle the important issue of fake or misleading reviews” (European Commission, 2016a, p. 8). This interpretation, however, does not seem to fit the literal meaning of the exceptions to the exemption of liability (Cauffman, 2016, p. 238). Fortunately, the recent proposal for a Digital Services Act intends to improve and clarify this aspect by suggesting that activities related to detecting illegal content (such as fake reviews) cannot preclude the application of the exemption of liability (Article 6).

#### The Unfair Commercial Practices Directive

The Unfair Commercial Practices Directive, on the other hand, states that online platforms or businesses must provide truthful information about the main characteristics of their services (see Articles 6 and 7, which, respectively, prohibit misleading actions and misleading omissions). When in place, online review mechanisms are an integral part of the services provided by online platforms or businesses. In this sense, professional parties must inform consumers about aspects such as how reviews are processed, how reviews are presented to consumers (including ranking criteria or recommendation systems), or how representative or accurate reviews are. The prohibition to omit material information means that, for example, the online platform cannot imply that online reviews are recent if they are over several years old. In fact, if platforms do not inform consumers on the potential (un)reliability of reviews (such as whether positive reviews are intentionally ranked higher than negative reviews), it might lead consumers to trust them and to make transactional decisions that they would not otherwise have taken. This would constitute a misleading omission and, therefore, an unfair commercial practice (Articles 5–7). An unfair commercial practice might lead to penalties of a maximum of (at least) 4% of the trader’s annual turnover (Article 13(3), introduced by Directive 2019/2161/EU). It is noteworthy, however, that this provision has a limited scope of application, namely that of Article 21 of Regulation (EU) 2017/2394 (concerning enforcement measures in coordinated actions).

The Unfair Commercial Practices Directive is the best legislative instrument at EU level to deal with unreliable reviews, due to its broad prohibition to omit material information and to its imposition to reveal material information. However, this broadness also means that its application to online review–related practices will not be completely established until courts explicitly interpret it that way. This interpretation has been pushed by the European Commission, which has also promoted the role of the Unfair Commercial Practices Directive in tackling online reviews’ unreliability in its Guidance Document on the Application of this Directive (European Commission, 2016b). There, the Commission provides several examples of online reviews–related practices that may be considered misleading, such as the screening, suppression, or prohibition of negative reviews. However, the regulation of review mechanisms offered by the Unfair Commercial Practices Directive is based mainly on the imposition of (indirect) information duties, which can be easily circumvented. As will be explained, the same approach was followed by the amending Omnibus Directive.

#### The Omnibus Directive

The Omnibus Directive entered into force in January 2020 and Member States must transpose it by May 2022. The Omnibus Directive aims at modernizing consumer rights, mainly by adjusting consumer rights to the platform economy and to the Digital Single Market. In this Directive, the EU legislator explicitly acknowledges the importance of online review mechanisms in the everyday life of a consumer (Recital 47). Concerning the regulation of online review mechanisms, the EU legislator, unsurprisingly, followed an information-regulation approach over a substantive one: For example, traders must inform consumers about the level of reliability of the review mechanism they operate, but they are not obliged to put any measures in place to effectively increase reliability. This information-based approach materializes in an addition to Article 7 of the Unfair Commercial Practices Directive. Newly added Article 7(6) prescribes that information on whether online reviews of products presented on the trader’s website originate from real consumers (that is, consumers who have purchased or used the product) as well as information on how the trader ensures that online reviews of products come from (real) consumers is considered material information. The fact that this information is explicitly considered material will determine that an omission of this information will most likely constitute an unfair commercial practice. The classification of a commercial practice as unfair also depends, of course, on other criteria, such as the factual context surrounding it, the limitations of the communication medium and, mainly, whether the average consumer is likely to have taken a different decision had he been informed about this material information (see Article 7(1) of the Unfair Commercial Practices Directive). In any case, these new provisions create additional indirect information duties for the trader operating an online review mechanism.

There are apparent exceptions to this information-based approach. The Omnibus Directive adds three new practices to Annex I of the Unfair Commercial Practices Directive that are considered unfair in all circumstances. Accordingly, these three practices concern the statement that the online reviews posted are submitted by real consumers without taking reasonable and proportional steps to guarantee the origin of those reviews; the submission of false reviews or endorsements; and the commission of another person to submit false reviews or endorsements. While the latter two practices indeed correspond to substantive protection (since they prohibit certain behaviours regardless of the information disclosed to the consumer), the first practice is more information-based than it may initially appear. The first practice entails that if the online platform does not claim that online reviews are submitted by real consumers, it does not have to take steps to guarantee their origin. Once again, it boils down to what information professional parties are conveying to consumers. If, on the other hand, the online platform states that reviews come from real consumers, it must put in place measures that guarantee it. Otherwise, that practice will be considered unfair.

On the one hand, the measures introduced by the Omnibus Directive regarding online review mechanisms partly match the recommendations made (and the expectations set) by the different studies on the role of online marketplaces conducted in the last couple of years at the European Commission’s request (for a detailed summary of these studies, see Narciso, 2019, p. 564). In fact, the main conclusion to extract from these studies was the appeal for online platforms to self-regulate and to put measures to increase reliability in place (e.g., EU studies suggested that online platforms apply a time-limited display of online reviews, European Commission, 2014a, p. 129). In this sense, not many legislative changes were to be expected. On the other hand, however, the new measures disappoint. The preparatory studies also recommended that the EU legislator puts in place measures to encourage a higher number of consumers to leave more reviews, which has not happened (European Commission, 2018, p. 56). Such a measure would increase the reliability of online reviews by imposing a bigger representation of consumer opinions. Then, what I have said elsewhere remains true: The *ratio* of the existing legal rules applicable to online reviews—to ultimately render online reviews a more reliable source of guidance—“can generally [and easily] be circumvented by merely informing consumers of how (un)reliable the review mechanism is” (Narciso, 2019, p. 580). Even if the legislator insists on an information-based approach, more could have been done. For example, imagine imposing the duty to inform consumers of the lack of representativeness of online reviews (e.g., “only 40 out of 1400 consumers have reviewed this product”). Overall, the amendments brought by the Omnibus Directive do not significantly add to the protection already granted by the Unfair Commercial Practices Directive, but they do add to legal certainty and predictability since now explicit references to online review mechanisms are made. In other words, the Omnibus Directive’s main added value is the materialization of the broad information duties imposed under the Unfair Commercial Practices Directive.

### Standardization and Soft Law

The regulation of online review mechanisms goes beyond (EU and national) legislation. In 2018, the International Standards Organization (ISO) published a standard on online consumer reviews (ISO 20488:2018), which was developed also mainly due to reliability concerns. In particular, the ISO highlights that the standard is meant to tackle false reviews written to mislead consumers, false reviews written to hurt competitors, blackmail of traders by consumers threatening to write a negative review unless they get a reward, misleading processing of reviews (like deleting negative reviews), and contracting consumers out of writing a negative review. The ISO sets a broad scope and clarifies that it applies to any organization that publishes online reviews, whether that is a professional party that collects reviews from their customers or an independent third party. The ISO standard defines online reviews as “any recorded information made publicly available by a consumer about a specified product or service or sold by a supplier.” At EU level, there is no official definition of an online review or of an online review mechanism, which contributes to some unclarity regarding the scope of application of EU consumer law to these mechanisms. Online review mechanisms can be part of not only different business models (such as platforms that offer reviews as their main activity—e.g., TripAdvisor—or businesses that offer it as a secondary activity—e.g., IKEA) but also derive from different parties. On this, see above the distinction between user reviews and professional reviews. In this sense, the existence of a definition in the ISO standard is beneficial, as it provides a starting point to acknowledge and better deal with the existing heterogeneity of feedback mechanisms. The standard imposes seven guiding principles: integrity, accuracy, privacy, security, transparency, accessibility, and responsiveness. Overall, organizations must guarantee the legitimate origin of the review, must have anti-fraud mechanisms in place, and should disclose “all methodologies (…) that may impact or influence a consumer’s use of the review site.” These guidelines are encompassing, clear, and, most importantly, include both a protection-through-information approach and a substantive (or interventive) approach, requiring that organizations inform consumers but also that they have in place concrete mechanisms to fight unreliability.

Additionally, the European Law Institute (ELI) contributed to the regulation of online reviews in consumer law by drafting a report on “Model Rules on Online Platforms” (henceforth “Model Rules”), adopted in February 2020 (European Law Institute, 2019). Initiatives like these are not infrequent in EU private law and are seen as creating soft law since they not only instigate academic debate but are often taken up as guidelines by EU associated institutions. The same process happened with the Principles of European Law (Mak, 2020, p. 144). The ELI Model Rules suggest several measures aimed at guaranteeing a minimum of transparency and reliability of online review mechanisms, particularly in Articles 5, 6, and 7. Similar to existing European legislation, the Model Rules follow a regulation through information approach. This is visible in Article 5, where the Model Rules suggest that the platform in charge of a review mechanism must provide information regarding the method of collecting, processing, and publishing online reviews, in order to increase reviews’ transparency. This information-based approach is also reflected, for example, in Article 6(h), according to which online platforms should inform users about factors impacting the consolidation of ratings based on reviews. It is in principle allowed for online platforms to develop ratings according to criteria other than the reviews’ or ratings’ numerical average, but those criteria must be communicated to consumers. The Model Rules also present substantive limits to this: The results of the consolidation cannot be misleading, and if reviews are displayed for a limited period, reviews over that period cannot be considered when aggregating them. Moreover, the Model Rules suggest substantive measures by stating, in Article 5(2), that reputation systems must comply with the requirements of professional diligence. A presumption of compliance would arise if, among other aspects, the platform took reasonable and proportionate steps to guarantee that the review is based on a real experience by the reviewer (Article 6). Similar to the ISO standard, the ELI presents a useful definition of a reputation system as “any mechanism for collecting and publishing reviews regarding suppliers, customers, goods, services or digital content” (Article 2(k)). Most importantly, the ELI model rules suggest how to connect the ISO standard on online reviews with existing EU consumer law: According to Article 5(3)(a), businesses who comply with this standard would be presumed to act according to professional diligence, which is a requirement also mentioned under Article 5 of the Unfair Commercial Practices Directive. This suggestion feels natural, and there is no reason for courts not to interpret Article 5 in this sense when looking at a review-related commercial practice.

### Self-regulation

In several studies preceding the Omnibus Directive, the EU has called for self-regulatory efforts by online platforms or businesses in charge of online review mechanisms. In principle, the economically rational behaviour for online platforms is the development of “honesty” mechanisms: the more honest and reliable reviewers are, the better the online platforms’ reputation is. This is a clear benefit of regulation by private actors: Private actors have, in this case, an incentive to fight deceptive information. However, while this seems to point towards the encouragement of self-regulation, it is important to note that “honesty” mechanisms do not always exist. This depends highly on the business model adopted by the platform. If the platform is not interested in the consumer choice of a specific product, it works (think again, for example, of TripAdvisor). If, on the other hand, the entity in charge of the review mechanism is the business-retailer itself, then there are incentives to, for example, omit negative reviews (for instance, business models like those of IKEA, H&M, or any restaurant).

Still, while EU consumer law’s regulation of unreliability in online review mechanisms is mainly based on the indirect imposition of information duties, platforms seem to have incorporated substantive reliability mechanisms. In its terms and conditions, Amazon states that it has a “zero tolerance policy for any review designed to mislead or manipulate customers.”^8^ In addition, Amazon’s terms and conditions inform that false, misleading, and manipulative reviews are not allowed. In particular, to prevent this, Amazon states that it will not accept reviews from a seller on a competitor’s product. This intolerance manifests on concrete sanctions: If Amazon’s guidelines are breached and if misleading or fake reviews are written, Amazon warns that it might restrict the infringer’s ability to use the platform or even terminate their account. TripAdvisor follows the same approach, and it informs that each review goes through a meticulous process to assess its veracity and reliability. In particular, TripAdvisor uses “smart technology” to look for data and patterns that indicate the presence of dishonest reviews. Besides, TripAdvisor has a 24/7 working team that makes decisions on the integrity of suspicious reviews. TripAdvisor revealed that in 2018 almost 3 million reviews were checked by its team (TripAdvisor, 2019, p. 6). On top of this, TripAdvisor allows users to flag suspicious reviews. These reviews are subsequently checked by the content moderation team. Writing a fake review or an intentionally misleading review may lead to the termination of the user’s account, as well as to reputational penalties (such as a reduction of the business’s standing in the popularity ranking). If the user persistently writes fake reviews, it can also face a content ban. Besides, TripAdvisor actively cooperates with public authorities. In 2018, TripAdvisor contributed to a judgement whereby the criminal court of Lecce (Italy) declared that writing fake reviews under a fake identity constitutes fraud under Italian criminal law. In this case, the fake reviewer was sentenced to nine months in prison and to pay 8,000 euros in damages.^9^

Amazon and TripAdvisor are not isolated in their active efforts to fight unreliable reviews. Indeed, other major online platforms have progressively improved the reliability (and transparency) of online review mechanisms. Illustratively, a few years ago, Booking.com added a feature to verify reviews (by having their staff individually read each review and approve it) and by archiving online reviews older than 36 months. As a response to consumer feedback on its review services, Trustpilot altered its procedure when it comes to reviews flagged as suspicious by consumers (Trustpilot, 2021, p. 35). Other reliability-related issues could be tackled through market design by platforms, such as better reflecting the needs of the consumer by recommending reviews written by users with similar needs (Edelman, 2017, p. 645). These examples show that even if EU legislation leaves some gaps and provides consumers mainly with information rights, this protection is complemented by concrete actions and sanctions by online platforms.

### *The Role of National Consumer Authorities*

National consumer authorities have also been active in fighting unreliable online reviews. In May 2015, the Danish Consumer Ombudsman published “Guidelines on the publication of user reviews,” which highlight the need for reliable consumer information and for transparent practices in the submission, processing, and posting of online reviews. In particular, the Danish Consumer Ombudsman explains how Danish law applies to online reviews, in an effort to increase compliance. A similar path was followed in 2016 by the Norwegian Consumer Authority (CA), who issued a report explaining how to apply national consumer law (particularly marketing law and the Norwegian Marketing Control Act from 2009, which implements the EU’s Unfair Commercial Practice Directive) to misleading reviews. The CA highlighted the need for uniform approaches to misleading reviews across jurisdictions. In May 2020, the UK’s Competition and Markets Authority (CMA) launched an investigation into fake and misleading reviews.^10^ The CMA highlighted the importance of such control given the increase in online shopping during the COVID-19 lockdown. The CMA had also previously carried work with selected platforms to improve review-related practices, and it had published a comprehensive report on the role of online reviews, providing advice for business compliance (2015). In 2017, the Dutch Authority for Consumers and Markets (ACM) conducted a study on online reviews and suggested that measures promoting transparency were added to the existing regime, as a way to increase the reliability of online reviews. In 2020, the ACM announced that it would be taking action against traders who present fake reviews and characterized them as “misleading endorsements,” while highlighting the need for consumers to be given “correct information.”^11^ Lastly, the ACM announced that it will admonish businesses, social media influencers, or other market players that facilitate “misleading endorsements.” If these entities refuse to stop their illegal activity, the ACM may even impose fines.

## Conclusions

It is unquestionable that unreliable online reviews have the potential to severely mislead consumers and to distort the internal market. In this sense, online review mechanisms need regulatory supervision. However, online reviews have, themselves, regulatory benefits to offer, particularly considering the failures in the EU law regulation of pre-contractual information in consumer contracts. The unreliability of online reviews has been pointed out as a reason to rule out this regulatory role, but this paper shows that consumers are reasonably protected against misleading or fake reviews, not only by the EU legislator but also by other regulatory actors. In fact, all the regulatory actors mentioned in this paper address online reviews’ unreliability, which shows that there is a multi-level approach to the issue. Most importantly, online platforms have, in general, an economic incentive to implement monitoring mechanisms, particularly if the proposed EU measures on the clarification of the exemption of liability under the E-Commerce Directive are adopted.

The existing legislative protection is still incomplete and could be improved. For example, some issues could be further addressed by the EU legislator, such as creating more incentives for platforms to self-regulate and, in specific, for platforms to encourage more consumers to review. Additionally, many broad legislation–based duties have just been introduced and their interpretation by the courts is still unclear. Nevertheless, consumers have several EU law–based rights against fake reviews. Put together, the rules imposed by EU consumer law, the work of national authorities, self-regulatory measures by the platforms, and the growing consumer awareness regarding the potential unreliability of online reviews show that, while the problem is not solved (and it will likely never be), enough protection has been granted to consumers so that we can consider reviews’ roles in the regulation of consumer information. From this perspective, online reviews can play a bigger role in consumer law than they currently do. Consumer law should not merely look at online reviews as an object of regulation, but also as a source of (inspiration for the) regulation of consumer information. This requires, naturally, further research, and this paper is intended as a stepping stone. This paper’s main assertion is that online reviews’ unreliability should not be used as a peremptory argument to deny them a larger role in the regulation of consumer information. In any case, a realistic regulatory goal can never be that of rendering reviews completely reliable or accurate. Not only is that against their nature—online reviews are, after all, valued for their subjective and personal character—it goes against human nature, since biases are inherent to decision-making.

## Footnotes

1 See the study conducted in 2019 by Which?, a UK’s consumer organization, see https://www.which.co.uk/news/2019/04/thousands-of-fake-customer-reviews-found-on-popular-tech-categories-on-amazon/ (accessed 28 June 2021).

2 Brightlocal, “Local consumer review survey 2020”, see https://www.brightlocal.com/research/local-consumer-review-survey/ (accessed 28 June 2021).

3 About buying and selling fake reviews and how that is becoming a problem for businesses, see, for example, the following news articles by the BBC: “Fake five-star reviews being bought and sold online” , seewww.bbc.com/news/technology-43907695 (accessed 6 July 2021); “Fake Amazon reviews 'being sold in bulk' online”, see https://www.bbc.com/news/business-56069472 (accessed 6 July 2021); “UK businesses caught buying five-star Google reviews”, see https://www.bbc.com/news/technology-56321576 (accessed 6 July 2021).

4 See the Financial Times’ news article “Amazon deletes 20,000 reviews after evidence of profits for posts”, see https://www.ft.com/content/bb03ba1c-add3-4440-9bf2-2a65566aef4a (accessed 6 July 2021).

5 See https://www.tripadvisorsupport.com/hc/en-gb/articles/204291188-Guidelines-for-Review-Helpful-Votes (accessed 6 July 2021).

6 See, for example and among many, BBC’s Kevin Peachey’s article on “Online reviews ‘used as blackmail’”, seehttps://www.bbc.com/news/business-33184207 (accessed 6 July 2021).

7 See www.booking.com (accessed 12 November 2021). While there is no direct link to access Booking.com’s guidelines on online reviews, it is possible to access them by clicking on any accommodation’s review section.

8 See https://www.amazon.com/gp/help/customer/display.html?nodeId=G3UA5WC5S5UUKB5G (accessed 6 July 2021).

9 See https://www.tripadvisor.co.uk/TripAdvisorInsights/w4237 (accessed 6 July 2021).

10 See https://www.gov.uk/cma-cases/online-reviews (accessed 5 July 2021).

11 See https://www.acm.nl/en/publications/acm-takes-action-against-trade-fake-reviews-and-fake-likes (accessed 5 July 2021).
